# Assessment of physicians’ resilience level during the COVID-19 pandemic

**DOI:** 10.1038/s41398-021-01395-7

**Published:** 2021-05-12

**Authors:** D. Douillet, A. Caillaud, J. Riou, P. Miroux, E. Thibaud, M. Noizet, M. Oberlin, M. Léger, R. Mahieu, E. Riquin, F. Javaudin, F. Morin, T. Moumneh, D. Savary, P.-M. Roy, O. Hugli

**Affiliations:** 1 Emergency Department, CHU Angers, Angers, France; 2 UMR (CNRS 6015—INSERM 1083) et Institut MitoVasc, UNIV Angers, Angers, France; 3 Micro et Nanomedecines Translationnelles, MINT, UNIV Angers, UMR INSERM 1066, UMR CNRS 6021, Angers, France; 4 Methodology and Biostatistics Department, Delegation to Clinical Research and Innovation, CHU Angers, 49100 Angers, France; 5 Emergency Department, Hôpitaux Civils de Colmar, Colmar, France; 6 Emergency Department, Centre Hospitalier de Mulhouse, Mulhouse, France; 7 Emergency Department, University Hospital of Strasbourg, Strasbourg, France; 8 INSERM, UMR 1260, Regenerative Nanomedicine, FMTS, Strasbourg, France; 9 Anesthesiology and Intensive Care Department, CHU Angers, Angers, France; 10 Department of Infectious Diseases, CHU Angers, Université d’Angers, Angers, France; 11 Univ Angers, Université de Nantes, Inserm, CRCINA, SFR ICAT, Angers, France; 12 Child and Adolescent Psychiatry Department, CHU Angers, Angers, France; 13 Department of Emergency Medicine, University Hospital of Nantes, Nantes, France; 14 EHESP, Irset, Inserm, UMR S1085, CAPTV CDC, Université Rennes, Rennes, France; 15 Emergency Department, Lausanne University Hospital, Lausanne University, Lausanne, Switzerland

**Keywords:** Genetics, Molecular neuroscience

## Abstract

We aim to assess physicians’ level of resilience and define factors that improve or decrease the resilience level during the COVID-19 pandemic. Physicians from hospitals located in areas with different COVID-19 caseload levels, were invited to participate in a national e-survey between April and May 2020. Study participants were mainly emergency physicians, and anaesthesiologists, infectious disease consultants, and intensive care. The survey assessed participant’s characteristics, factors potentially associated with resilience, and resilience using the Connor-Davidson Resilience Scale (RISC-25), with higher scores indicative of greater resilience. Factors associated with the resilience score were assessed using a multivariable linear regression. Of 451 responding physicians involved in the care of COVID-19 patients, 442 were included (98%). Age was 36.1 ± 10.3 years and 51.8% were male; 63% worked in the emergency department (*n* = 282), 10.4% in anesthesiology (*n* = 46), 9.9% in infectious disease department (*n* = 44), 4.8% in intensive care unit (*n* = 21) or other specialties (*n* = 49). The median RISC-25 score was at 69 (IQR 62–75). Factors associated with higher RISC scores were anesthesia as a specialty, parenthood, no previous history of anxiety or depression and nor increased anxiety. To conclude, this study is the first to characterize levels of resilience among physicians involved in COVID-19 unit. Our data points to certain protective characteristics and some detrimental factors, such as anxiety or depression, that could be amenable to remediating or preventing strategies to promote resilience and support caregivers in a pandemic.

## Introduction

The upheavals induced by the pandemic linked to SARS-CoV-2 infections have historical proportions. Many hospitals worldwide have faced of a surge in patients with COVID-19, while others have been planning for it and reorganizing their entire operations to avoid being overwhelmed^1,2^. These new processes have involved increased bed capacity in ICUs and wards, separate patient streams, adoption of new technologies and communication systems, staff reassignment, and the reorganization of physical spaces^3^. Healthcare providers had to adapt to abrupt changes to their working conditions, and had to deal with new colleagues, unfamiliar working space, ever changing personal safety and treatment guidelines, while facing shortages of personal protective equipment, medications, and ventilators. They have cared daily for severely ill or dying patients on a daily basis, some of them their colleagues, while facing the risk of their own infection.

These dramatic events have highlighted the importance of resilience^4^. Resilience is the subject of growing interest in the fields of psychiatry, psychology, sociology, and economics. Resilience is an evolving concept, defined as the “resources as positive psychological, behavioral, and/or social adaptation in the face of stressors and adversities^5^”. Compared with nonmedical health workers, medical health workers have experienced a significantly higher prevalence of insomnia, anxiety, depression, somatization, and obsessive-compulsive symptoms^6,7^. In a Chinese multicentre survey of physicians, the prevalence of depression was 50.7%, anxiety 44.7%, insomnia 36.1%, and stress-related symptoms 73.4%^8^. However, after disasters, most people are resilient and do not develop long-lasting mental disorders . Although the negative effects of the current crisis on physicians’ well-being have been studied^6,8,9^, few studies have assessed “the force within everyone that drives them to seek self-actualization, altruism, wisdom, and harmony with a spiritual source of strengths,” namely resilience^10^. Wellness incorporates mental, physical, and spiritual health to protect against burnout. The primary aim of this study was therefore to assess physicians’ level of resilience and define factors that improve or decrease their resilience level.

## Materials and methods

### Design

We conducted a cross-sectional study between April 18 and May 10, 2020 that assessed physicians’ resilience during the COVID-19 pandemic in several French hospitals.

### Participants and settings

#### Participants

Physicians of six initial centers were selected to participate in this study. Study participants were mainly emergency and intensive care physicians, anesthesiologists, and infectious disease specialists. We used a snowball sampling method^11^, i.e., the initial invitation to the physicians of the six participating centers stated that it was possible (but not obligatory) to disseminate the survey to colleagues in the same speciality. Only board-certified physicians could participate; therefore, excluding residents.

Participation was voluntary, and signed consent was not requested. Filling out the questionnaire was considered implicit proof of consent. No incentive was offered. Data were collected anonymously.

#### Assessment of caseload according to centers

The initial sample of six hospitals was selected according to their real caseload from each of three regions (low, moderate, high level of real caseload). To do this, we used the national real-time data published by the French Ministry of Health on COVID-19^12^. This caseload was mainly determined by region according to the proportion of usually open resuscitation beds that were then occupied by COVID-19 patients: high caseload above 60%, intermediate caseload between 40 and 60%, and low caseload between 0 and 40%. The centers of Angers and Nantes had a low, Cahors and Paris an intermediate, and Mulhouse and Colmar a high caseload.

#### Development and pretesting

Participants completed a 41-question survey specifically designed for this study. The questionnaire had three distinct sections: participant characteristics (5 questions), factors potentially associated with resilience (11 questions), and finally a resilience scale using the 25-item French version of the Connor-Davidson Resilience Scale (CD-RISC 25)^13^. The scale explores seven domains of resilience: hardiness (i.e., commitment/challenge/control), coping, adaptability/flexibility, meaningfulness/purpose, optimism, regulation of emotion and cognition, and finally self-efficacy^13^. Each of the 25 items is rated on a 5-point scale (0–4), with a possible total score range from 0 to 100 points, with higher scores indicative of a greater resilience. In the US general population, from whom this score was derived, the median score was 82 points, with quartiles being 0–73, 74–82, 83–90, 91–100^13^. The scale has since been validated in the general population^14–16^, among patients with post-traumatic stress disorder^17^, and among healthcare workers^18–20^. Sensitive questions were asked, e.g., concerning recreational drug use, for which reason the survey was anonymous to guarantee the veracity of answers. In order to assess the factors associated with different levels of resilience and not overburden participants, anxiety requiring treatment, depression under treatment, stress and alcohol or tobacco consumption have been only assessed in a declarative way. Physicians were asked to assess subjectively their perceived caseload of patients with COVID-19 using a 5-level Likert scale (0: no caseload, 1: very low caseload, 2: low caseload, 3: normal caseload, 4: high caseload, 5: very high caseload). The questionnaire was pretested on a small sample of ten physicians before fielding the survey.

#### Survey administration

In each center, a local investigator sent a personal invitation email to the different specialists (mainly emergency physicians, intensivists, anesthesiologists, and clinical infectious disease consultants). The email contained a link to the online self-administered questionnaire. The questionnaire was posted on Google Forms^®^. The original investigators had the possibility of transferring this survey to their contacts in the same speciality. The survey was open, and to prevent multiple entries, we compared the participants’ characteristics and if similar, their questionnaire would have been removed. However, no duplicate questionnaire was found. The response rate could not be calculated, because of the snowball sampling methodology. A reminder was sent after 10 days to all local investigators.

The specific objective of this study, i.e., the measure of resilience, was not initially explained to participants, who were only informed that the e-survey addressed their mental health.

### Analysis

Only completed questionnaires were analyzed. The Checklist for Reporting Results of Internet E-Surveys was followed^21^.

Continuous variables were summarized as mean and standard deviation, or median values with interquartile ranges, while categorical variables were reported as counts and percentages. Continuous variables were compared using the Mann–Whitney *U* test or the Kruskal–Wallis test, and categorical variables were compared using the Chi-square test. A bilateral *p* value < 0.05 indicated statistically significance. We performed a univariate analysis to select the predictor variables associated with higher level of resilience by using the Chi-square test. We then performed a multivariable linear regression with a backward stepwise elimination, initially including all variables associated with the CD-RISC-25 score with a *p* value < 0.2 in the univariate analysis. We verified the absence of collinearity between the explanatory variables. All data were analyzed using R (R Core Team, 2014, R: a language and environment for statistical computing. R Foundation for Statistical Computing, Vienna, Austria).

### Ethics

This study was approved by the Ethics Committee of the Angers University Hospital (A 2020–30) and declared on clinicaltrials.gov before inclusion of the first participant (NCT04349163).

## Results

A total of 451 physicians caring for COVID-19 patients returned a fully completed questionnaire, of which 9 (2.3%) were subsequently excluded: 1 as the respondent was a pharmacologist not directly involved in patients’ care, 4 were residents, and 4 contained errors. Of 442 valid respondents, mean age was 36.1 ± 10.3 years and 213 (48.2%) were female (Table 1). Regarding the medical specialty, 282 were emergency physicians (63.3%), 46 anesthesiologists (10.4%), 44 infectious disease specialists (9.9%), and 21 intensive care specialists (4.8%). The remaining 49 physicians (11.1%) were from several other specialties (geriatric, pneumology, internal medicine, dermatology, cardiology, general medicine, etc.), dispatched to work in COVID-19 units. Almost all physicians were working full time (94.4%). According to the real caseload based on French national data, 65.2% of physicians were in a low (*n* = 288/442), 2.3% in a moderate (*n* = 10/442), and 32.5% in a high caseload area (*n* = 144/442). According to the physicians’ perceived caseload, 132 physicians (29.9%) considered to face a low caseload, 218 (49.3%) a normal caseload, and 91 (20.6%) a high caseload. The physician’s perception was not significantly discordant with national data. The median postgraduate training was 8 years (3–17). With regard to familial status, 71 (16.1%) were single and 371 (83.9%) lived with a partner; 267 (60.4%) had children. Almost all physicians were under curfew at home with their spouse or family (375/442, 84.8%). The others (15.2%) were alone or in another place, or with friends or relatives. Half of them were afraid of infecting their relatives (223/442, 50.5%) with the coronavirus. Few physicians reported increased tobacco use (20/442, 4.5%) or both tobacco and alcohol use (15/442, 3.4%), while significantly more increased their alcohol consumption (92/442, 20.8%).

**Table 1:** Demographic characteristic of the study population.

Characteristics	Total *n* = 442 (%)
Male sex	229 (51.8)
Age, mean, y (SD)	36.1 (10.3)
Physicians specialty	
Emergency medicine	282 (63.3)
Anesthesiology	46 (10.4)
Infectious disease	44 (9.9)
Intensive care medicine	21 (4.8)
Others	49 (11.1)
Full-time equivalent, median % (IQR)	94.4 (92–100)
Caseload according to physician gestalt^a^	
None or low (0,1)	132 (29.9)
Normal (2)	218 (49.3)
High or very high (3,4)	91 (20.6)
Years of experience (IQR)	8 (3–17)
Family situation	
Single without children	52 (11.8)
Single with child(ren)	19 (4.3)
Couple without children	123 (27.8)
Couple with child(ren)	248 (56.1)
Type of quarantine	
At home alone	53 (12)
At home with spouse or family	375 (84.8)
At home with another person (friend, roommates…)	13 (2.9)
In another location	1 (0.2)
Fear of infecting relatives	223 (50.5)
History	
Anxiety	23 (5.2)
Depressive syndrome	11 (2.5)
Anxiety and depressive syndrome	20 (4.5)
Anxiolytic medication before the COVID-19 period	7 (1.6)
Anxiolytic medication during the COVID-19 period	18 (4.0)
Smoking before the COVID-19 period	67 (15.2)
Drug addiction before the COVID-19 period	3 (0.7)
Consumption of alcohol before the COVID-19 period	215 (48.6)
Increased anxiety	114 (25.8)
Increased tobacco consumption	20 (4.5)
Increased alcohol consumption	92 (20.8)
Increased tobacco and alcohol consumption	15 (3.4)

Based on a Likert scale.

^a^Based on the rate of hospitalization for COVID-19, the occupancy rate of intensive care bed, cumulated death rate in the hospital department.

**Fig. 1:**
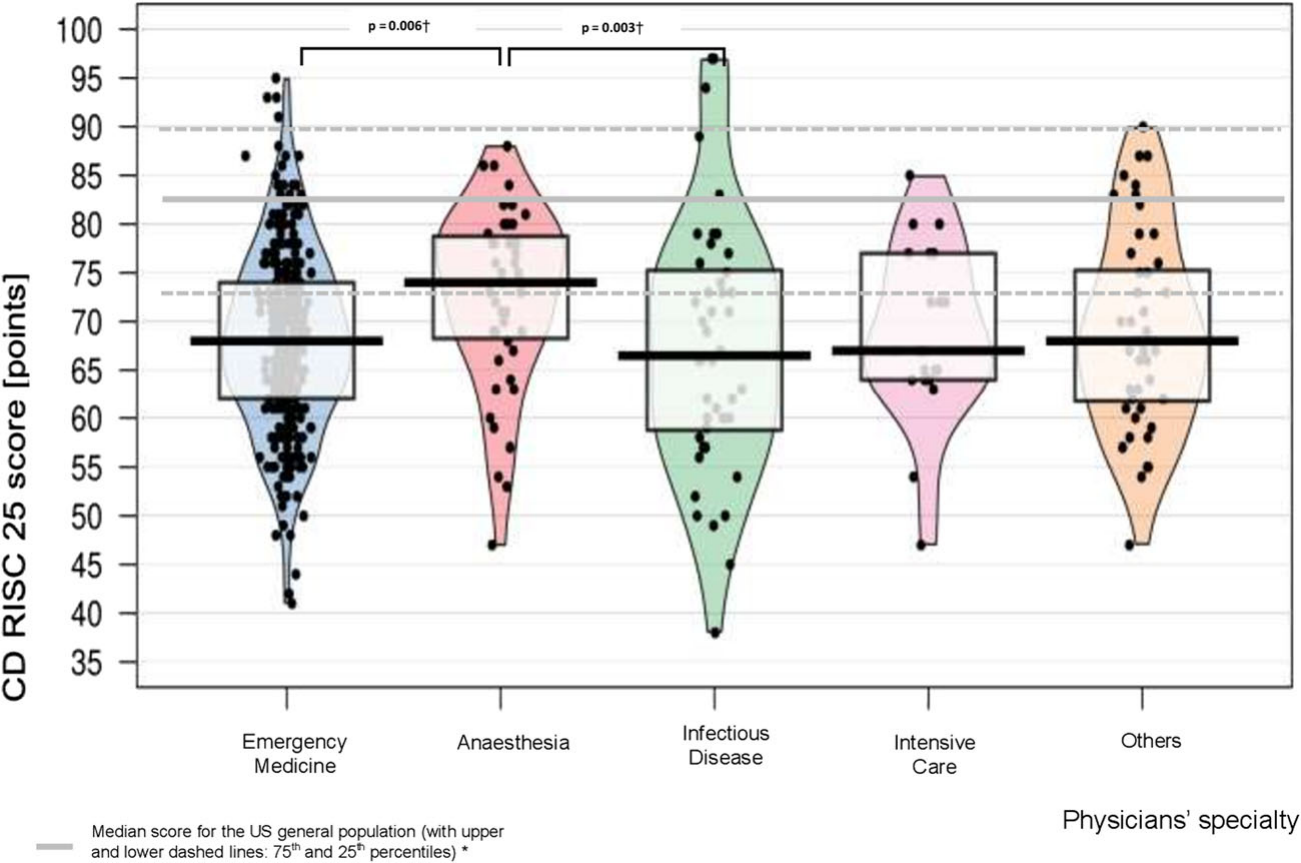
Violin plots according to the resilience’s scores and the kind of specialty. Resilience was assessed using the CD-RISC 25 scale [0–100]. In the box plots, the boundary of the box closest to zero indicates the 25th percentile, a black line within the box marks the median, and the boundary of the box farthest from zero indicates the 75th percentile. *Median in the US general population in the original description of the CD-RISC25 = 83 (73–90). ^†^Global comparison was performed using Kruskall–Wallis test (*p* = 0.02) and post-hoc test using Dunn test with a Hochberg multiple comparison procedure, p significant.

**Fig. 2:**
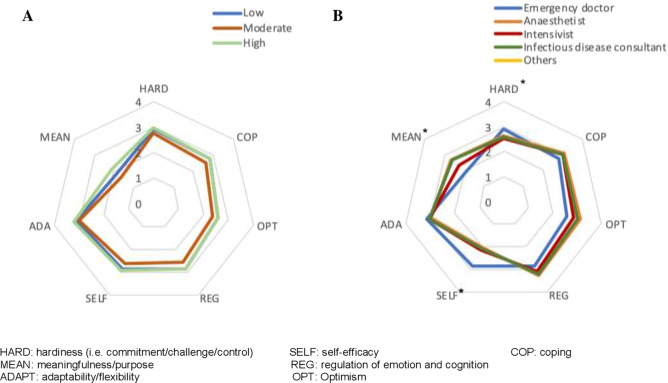
Average score of the seven components of the resilience score according to physicians’ characteristics. **A** Low, moderate, and high level of caseload. **B** Physician specialty.

The median resilience score was at 69 (IQR 62–75), and several factors were associated with higher RISC scores (Tables 2, 3 and Fig. 1): medical specialties in anesthesia, a high caseload according to the national data (but not according to physician’s gestalt), and parenthood. On the other hand, physicians with a self-reported history of anxiety, stress, and/or depression and physicians who experienced increased anxiety during the pandemic period had lower resilience scores (*p* < 0.05). No other demographic variables could be associated with resilience scores. The different subscales of resilience were similar across caseload levels, but statistically different between emergency physicians and other specialties (*p* < 0.05), with higher scores for self-efficacy and hardiness, and lower scores for meaningfulness (Fig. 2).

**Table 2:** Classification of levels of resilience measured by CD-RISC 25 grouped by a range of characteristics.

	Univariate analysis	
Characteristics	Resilience score^a^	*p* value^b^
Male sex	68 (62–75)	0.68
Female sex	69 (62–75)	
Age		0.10
<35 years	68 (62–75)	
35–55 years	68 (62–76)	
>55 years	71 (65–79)	
Physicians specialty	69 (62–75)	0.02
Emergency medicine	68 (62–74)	
Anesthesiology	72 (68–79)	
Infectious disease	67 (59–76)	
Intensive care medicine	69 (64–77)	
Others	69 (62–76)	
Full-time equivalent		0.12
Yes	68 (62–75)	
No	70 (64–76)	
Caseload according to physician gestalt^c^		0.32
None or low (0, 1)	69 (63–74)	
Normal (2)	68 (62–76)	
High or very high (3, 4)	69 (62–76)	
Caseload according to national data		0.05
Low	68 (41–75)	
Normal	63 (52–67)	
High	70 (63–76)	
Difference in caseload perception and reality		0.43
Less caseload perceived	68 (61–76)	
Concordance	69 (62–75)	
More caseload perceived	69 (63–75)	
Family situation		
Living with ≥child	69 (63–76)	0.02
Living without child	67 (60–74)	
Type of quarantine		
Quarantine with ≥1person	69 (62–75)	0.40
Quarantine alone	67 (61–75)	
Fear to contaminate relatives		0.72
Yes	68 (62–72)	
No	66 (63–71)	
History of anxiety/stress/depression		<0.01
Yes	63 (55–70)	
No	69 (63–76)	
Increased anxiety		<0.01
Yes	65 (59–72)	
No	67 (63–76)	
Increased tobacco and/or alcohol consumption		<0.01
Yes	66 (61–72)	
No	70 (63–76)	

ref reference.

^a^Resilience score is the median and the interquartile of the CD-RISC 25.

^b^Calculated with the Mann–Whitney *U* test or Kruskal–Wallis test with significantly threshold *p* value < 0.05 with multiple testing adjustment (Hochberg).

^c^According a Likert scale from 0 to 5, comparison between high level of caseload and other level of caseload.

**Table 3:** Multivariate analysis.

Variables	Regression coefficient^a^	95% Confidence interval	*p* value
Anesthesiology specialty (vs. others specialty)	1.9	1.1–4.6	0.03
High caseload level (vs. normal caseload level)	1.2	0.22–2.12	0.02
Living with ≥1 child (vs. no child)	1.8	0.03–3.6	0.05
History of anxiety/ stress/depression (vs. no history)	3.7	0.92–6.47	0.01
Increased anxiety (vs. no increased anxiety)	4.5	2.62–6.35	<0.01

^a^Multivariable linear regression, significantly threshold *p* value < 0.05. Only significant variables are presented. The initial model included all variables associated with the outcome at the *p* < 0.2 level in the univariate analysis, i.e.,: age, full-time equivalent, caseload according to national data, family situation (child or not), history of anxiety/ stress/depression, increased anxiety, increased tobacco, and/or alcohol consumption.

In the multivariate model, we included all variable significantly associated with higher level of resilience. The physician specialty (anesthesiology), parenthood, having no declared history of anxiety and/or depression, and no increased anxiety were associated with higher level of resilience during the COVID-19 crisis (Table 3).

## Discussion

The Resi-CoV study is one of the first studies to assess resilience among physicians taking care of COVID patients. We found that median total RISC score was 69 points, but the range was wide, spanning from 38 to 97 points. Based on our multivariable linear regression model, to be an anesthesiologists, parents, without a history of anxiety, stress, or depression or without increased anxiety during the pandemic period were factors associated with a higher overall RISC score. The average scores of the seven components of the RISC score did not differ based on the caseload levels but differed between medical specialties. Emergency physicians had higher self-efficacy and meaningfulness/purpose subscores, but a lower meaningfulness subscore.

With a median CD-RISC-25 score of 69, the resilience score of surveyed French physicians was lower than that found in other studies conducted in the general US population^13,22,23^, corresponding to the lowest quartile^13^. A few studies have assessed the level of physicians’ resilience since the beginning of the COVID-19 pandemic. Using the CD-RISC 25, in Wuhan, the resilience score was higher, at 73.48 ± 11.49^24^. Our results were also lower than those found among both inexperienced and experienced Chinese healthcare workers during the pandemic, with scores of 67.73 ± 14.85 and 75.36 ± 13.27, respectively^25^. Meynaar et al. found an increased prevalence of burnout among intensivists, which was inversely correlated to the resilience and work engagement scores^26^. Consistent results were found in Canada, and in Turkey: resilience was an important factor associate with reduced stress and distress during this COVID-19 crisis^27,28^.

However, as resilience is a process and not a stable trait throughout life, longitudinal data would be needed to measure the impact of the pandemic on population or individuals^5,29^.

One in four physicians in our sampled felt more anxious, one in five increased their consumption of alcohol, and one in twenty their tobacco consumption. Stress contributes to unhealthy behaviors^30,31^, in particular for those who are less resilient, and the long-term consequences of these behavioral changes will need to be reassessed. On the other hand, parenthood was significantly associated with higher levels of resilience^5^, pointing to the crucial contribution of a healthy work-family balance to the healthcare providers’ psychological well-being during this pandemic^32^. Indeed, being a parent could lead to overall well-being, a more positive emotional experience and meaning from one moment to the next^33^.

In this study, anesthesiologists had significant higher level of resilience. Stress is inherent to their daily practice, against which they may have developed psychological coping mechanisms^34^. However, they tend to be more prone to burnout than other physicians^35,36^. Emergency physicians were the most represented specialists in our study and showed a lower level of overall resilience. A recent study showed that the personality of residents in emergency medicine differed considerably from that of other physicians and, in response to stress, they may become risk averse^37^. On the other hand, residents in emergency medicine scored higher than other specialists in the self-efficacy and hardiness and lower in meaningfulness^37^. These results are consistent with our study finding conducted in certified emergency physicians.

No significant difference was found between the level of tension in centers. This can be explained by the fact that all the emergency departments needed a major reorganization in preparation for the surge of patients with COVID-19.

Using the CD-RISC, Mealer et al. showed that older age was significantly associated with high level of resilience among ICU nurses^20^. In a cross-sectional study in the UK National Health Service, a weak positive correlation between age and resilience was found among older employees displaying a higher level of resilience^38^. However, in our study, older respondents tended to score higher, although the difference failed to reach statistical significance.

Should low resilience among emergency physicians be the focus of an intervention program, beyond the current COVID-19 crisis? Resilience is a key component of maintaining personal health and quality of care in the workplace, despite adverse life events^39–41^. According to a meta-analysis based on cross-sectional studies, greater resilience is associated with less depressive symptomatology^42^. Many programs exist to improve resilience^43,44^, and some strategies may support health professionals’ resilience. The Ontario Medical Association—Physician Health Program suggests a series of ten practical steps to promote resilience during the COVID-19 crisis: from relying on basic notions of daily needs, in the management of friendly and family relationships up to rules of cohesion at work^45,46^. The French National Center for Resources and Resilience propose 11 steps for all people (not just caregivers): maintain the self-efficacy, tolerate uncertainty, increase our sense of security, remember the facts, let’s trust, be flexible when faced with the necessary adaptations, focus on activities that are good for well-being outside of your work, be kind to ourselves, look to the future with positive thoughts, stay in touch with friends and family, and increased our solidarity^47^. Team and individual debriefs are another method shown to decrease professional stress and improve concentration, morale, and commitment to work^48^. Debriefing meetings, as a team or individual during this difficult period, reinforce resilience to compassion fatigue^49,50^. During this period, department projects should be stopped to allow all the limited available time outside of care to be dedicated to healthcare workers’ relaxation, sleep, and family time^39^. In a recent letter, the benefit of online Balint group meetings on resilience, assessed by the CD-RISC 25 score, was suggested for a small group of healthcare providers caring for COVID-19 patients in Iran^51^. However, not all interventions have the same effectiveness^52^, and each program should be evaluated rigorously before implementation.

Resilience extends beyond individual healthcare providers, and applies to the intrinsic ability of hospitals and the healthcare system to cope during crises^53^. Resilience engineering, based on top management commitment, increased flexibility, learning lessons from both incidents, and normal operation and awareness of the system status, is an essential part of preparedness to face man-made or natural disaster^54^.

### Limitations

This study has several limitations. First, we included a large number of physicians involved in COVID patient care, relying on a snowball sampling methodology. The drawback if this approach is that we cannot know the participation rate, due to our lack of control over the email distribution list. Second, we found statistically significant differences between groups in the RISC score, but the minimally clinically significant difference on the RISC scale is unclear. Third, our sample comprises primarily emergency physicians, thus limiting the portability of our findings to other specialties. Fourth, respondents may differ from nonrespondents, and we cannot exclude a selection bias. Fifth, although the questionnaire was anonymous, some respondents may not have fully disclosed their behaviors or true level of resilience. Data concerning history of anxiety requiring treatment, of depression under treatment, of stress and alcohol or tobacco consumption were self-reported and are subject to recall or social desirability bias. Finally, although the CD-RISC score is well validated, resilience is a complex phenomenon and it is a process, not a trait^5^. Our survey provides a snapshot of the crisis and may not reflect a permanent trait in physicians caring for COVID-19 patients.

## Conclusions

Resilience varied among French physicians, and lower scores were associated with increased anxiety with potentially harmful behaviors. Parenthood is associated with a higher level of resilience. The COVID-19 outbreak is an opportunity to reaffirm the importance of caring for the caregivers.
